# Non-chemical Control of Root Parasitic Weeds with Biochar

**DOI:** 10.3389/fpls.2017.00939

**Published:** 2017-06-07

**Authors:** Hanan Eizenberg, Dina Plakhine, Hammam Ziadne, Ludmila Tsechansky, Ellen R. Graber

**Affiliations:** ^1^Department of Plant Pathology and Weed Research, Newe Ya’ar Research Center, Agricultural Research OrganizationRamat Yishai, Israel; ^2^Institute of Soil, Water and Environmental Sciences, Agricultural Research Organization, The Volcani CenterBeit Dagan, Israel

**Keywords:** biochar, tomato, root parasitic weeds, broomrape, ecological control, climate change mitigation

## Abstract

This study tested whether soil-applied biochar can impact the seed germination and attachment of root parasitic weeds. Three hypotheses were evaluated: (i) biochar adsorbs host-exuded signaling molecules; (ii) biochar activates plants’ innate system-wide defenses against invasion by the parasite; and (iii) biochar has a systemic influence on the amount of seed germination stimulant produced or released by the host plant. Three types of experiments were performed: (I) pot trials with tomato (*Solanum lycopersicum*) infested with *Phelipanche aegyptiaca PERS*. (Egyptian broomrape) and three different types of biochar at concentrations ranging from 0 to 1.5% weight, wherein tomato plant biomass, *P. aegyptiaca* biomass, and number of *P. aegyptiaca*-tomato root attachments were quantified; (II) split-root biochar/no-biochar experiments under hydroponic growing conditions performed in polyethylene bags with tomato plant rootings, wherein *P. aegyptiaca* seed germination percentage and radicle attachment numbers were quantified; and (III) germination trials, wherein the effect of biochar adsorption of GR-24 (artificial germination stimulant) on *P. aegyptiaca* seed germination was quantified. Addition of biochar to the pot soil (Experiment I) resulted in lower levels of *P. aegyptiaca* infection in the tomato plants, mainly through a decrease in the number of *P. aegyptiaca* attachments. This led to improved tomato plant growth. In Experiment II, *P. aegyptiaca* seed germination percentage decreased in the biochar-treated root zone as compared with the no-biochar control root zone; *P. aegyptiaca* radicle attachment numbers decreased accordingly. This experiment showed that biochar did not induce a systemic change in the activity of the stimulant molecules exuded by the tomato roots, toxicity to the radicles, or a change in the ability of the radicles to penetrate the tomato roots. The major cause for the decrease in germination percentage was physical adsorption of the stimulant molecule by the biochar (Experiment III). Adding biochar to soil to reduce infections by root parasitic weeds is an innovative means of control with the potential to become an important strategy both for non-chemical treatment of this family of pests, and for enhancing the economic feasibility of the pyrolysis/biochar platform. This platform is often viewed as one of a handful of credible strategies for helping to mitigate climate change.

## Introduction

The pyrolysis/biochar platform has enjoyed considerable attention in recent years because it has the potential to convert organic wastes into renewable energy, sequester atmospheric carbon, and improve soil fertility. Yet, despite the intense and continually expanding scientific and industrial interest in this platform, it is still in an immature state, in part because much remains to be learned regarding its potential for restoring soil health and functioning. While the addition of biochar to soils has, on average, a positive influence on crop growth and productivity, some systems demonstrate negative or no effect of soil-added biochar (Jeffery et al., 2011, 2017; Biederman and Harpole, 2013; Crane-Droesch et al., 2013). The mechanisms responsible for such inconsistent results are far from understood. Adding biochar to soil is known to create changes in the plant/root zone/soil system that can affect plant performance and health by virtue of a number of inter-related physical, chemical and biochemical processes (Graber et al., 2014a), including (i) nutrient supply and balance; (ii) soil pH and redox, (iii) soil structure and functioning; (iv) microbial community structure; (v) plant–microbe signaling; and (vi) release/sequestration of phytotoxic, biotoxic, or bioactive compounds.

Biochar-elicited changes at the soil/root interface have been found to play a role in activating plants’ innate defenses against disease-causing microbial pathogens along multiple hormone pathways (Meller Harel et al., 2012; Mehari et al., 2015). Frequently, impacts on both plant resistance to disease and on plant growth are related to biochar dose, with biochar exhibiting a hormone-like effect of low dose stimulation and high dose inhibition (Graber et al., 2014a; Jaiswal et al., 2015). Changes in microbial community structure, functioning and diversity caused by biochar additions are implicated in some of these effects (Kolton et al., 2017). One of the ways in which biochar can alter microbial community structure and dynamics is by adsorbing compounds involved in bacterial intercellular signaling (Masiello et al., 2013).

Generally, the adsorption ability of biochar greatly exceeds that of native adsorbing phases in soils (Graber and Kookana, 2015). Adding a strong adsorbent such as biochar to the soil thus can have considerable and varied impacts on the soil system. This has been documented with respect to soil contaminants, pest control products, and bacterial signaling molecules. The question arises whether soil-applied biochar can interfere with plant–plant or plant–bacteria communications, much the way it interferes with bacteria–bacteria signaling, via adsorption deactivation of signaling molecules.

One type of subsurface plant–plant interaction promoted by signaling molecules is germination of root parasitic weed seeds induced by chemicals released into the rhizosphere by the roots of the host plants (Yoneyama et al., 2010). Seed germination that relies on a host-specific signaling molecule precludes the possibility that the seeds will germinate in the absence of the host. This is crucial because the parasitic weed embryo will die if its radicle does not attach itself to a host root within a distance of only a few millimeters (Joel and Bar, 2013). Any process that interferes with host-parasite signaling, such as adsorption of the signaling molecule, or a change in the host production of the signaling molecule, could influence germination of parasitic weed seeds and hence, the intensity of infection.

To the best of our knowledge, the effect of biochar on weed growth has been studied only in autotrophic (Major et al., 2005; Adams et al., 2013; Soni et al., 2014) and hemiparasitic weed species (Smith and Cox, 2014). Neither of these types requires host-specific signaling molecules for seed germination. In contrast, root parasitic weed seeds can only germinate when they sense the presence of the host-exuded signaling molecules.

Lacking photosynthetic activity, root parasitic weeds rely wholly on the host plant for their germination, nutrition and water (Parker, 2013). They are a serious threat to many important crops, including tomato, potato, sunflower, canola, fava bean, pea, carrot and more. By and large, there are only a few effective treatment options for such weeds, and these options are neither economically viable nor ecologically sound. For example, *Phelipanche aegyptiaca* in open field tomatoes and *Orobanche cumana* in sunflower can be effectively controlled with herbicides delivered via drip irrigation system or by soil fumigation, but doing so requires advanced technologies (Eizenberg et al., 2012) and is expensive (700 to 7,000€ per ha). Today, no solutions for *P. aegyptiaca* management in organic farming and in greenhouse tomatoes are available, particularly in the Mediterranean basin (e.g., Turkey, Greece, Italy, Spain, Morocco and Israel), where infestation with *P. aegyptiaca* and *P. ramosa* is rampant (Parker, 2013). Both organic tomatoes and greenhouse tomatoes are attractive high cash crops for farmers.

The current study represents the first test of whether soil-applied biochar impacts the germination, attachment, and development of root parasitic weeds. *P. aegyptiaca* PERS. (Egyptian broomrape) in tomato (*Solanum lycopersicum*) was selected as the test case. Specifically, we evaluated three hypotheses, each of them involving a mechanism that could affect the extent of broomrape infection under biochar application: (i) biochar adsorbs host-exuded signaling molecules; (ii) biochar activates plants’ innate system-wide defenses against invasion by the parasite; and (iii) biochar has a systemic influence on the amount of seed germination stimulant produced or released by the host plant.

## Materials and Methods

### Biochars

Three types of biochar were tested: (i) biochar produced from wastes of greenhouse pepper plants in a modified slow pyrolysis unit (All Power Labs, San Francisco, CA, United States) at a highest treatment temperature (HTT) of c. 350°C, designated herein as GHW-350 (lab no. B8, GHW-350.5); (ii) biochar produced from the same wastes in the same unit at an HTT of c. 600°C; designated herein as GHW-600 (lab no. B9, GHW-600.2); and (iii) biochar produced from shredded date palm fronds using a home-made top-lit up-draft (TLUD) gasifier based on the ELSA design of Blucomb (Udine, Italy^1^) at an HTT of c. 650°C; designated herein as PALM-650 (lab no. D3, PALM-650.1). The two biochar feedstocks are common agricultural wastes in Israel and are candidates for pyrolysis solutions. These wastes are produced in the same geographical areas as are crops suffering from root parasitic weed infestations. Physical and chemical characteristics of the biochars [specific surface area (SSA), iodine number, inorganic matter content, solution pH, solution electrical conductivity, inorganic C, organic C, and element analysis] were determined according to methods in Graber et al. (2014b) and are reported in **Table 1**.

**Table 1:** Physical and chemical characteristics of the biochars.

Characteristic	Units	GHW-350	GHW-600	PALM-650
Specific surface area (SSA)	m^2^/g	1.3	2.9	9.0
Iodine number	mg/g	n.a.ˆ+	93	108
Mineral content (Ash)	%	61.0	77.5	63.2
pH^#^	Units	8.5	10.5	8.5
EC^#^	mS/s	7.9	4.2	4.1
C inorganic	%	4.1	6.7	2.2
C organic (C_org_)^∗^	%	44.0	24.4	62.9
H^∗^	%	3.3	1.1	2.2
N^∗^	%	3.5	1.6	1.4
S^∗^	%	1.7	1.0	1.0
H/C_org_	Molar ratio	0.90	0.55	0.42

*The biochars have high inorganic mineral contents because the feedstocks from which they originated were grown under intense fertilization and irrigation with saline water (3.5–4.5 mS/s). GHW refers to foliage and stems of greenhouse pepper plants, and PALM refers to date palm fronds. ^+^n.a. – not amenable to determination. ^#^pH and EC determined in 1:20 biochar:deionized water suspension. ^∗^C_*org*_, H, N, and S are reported on an ash-free basis.*

### Tomato-Broomrape Pot Trial

Tomato plants were grown in soil infested with *P. aegyptiaca* seeds and treated with various levels of different biochars to determine if the biochars had any effect on *P. aegyptiaca* attachment and biomass, and tomato plant development. Tomato (*S. lycopersicum*) cv. 4343 (non-terminated type; Adama, Israel) was used. *P. aegyptiaca* inflorescences were collected from a broomrape-infested tomato field (Mevo Hama, Israel). Prior to the pot trial, *P. aegyptiaca* seed germination rates were confirmed to be 84% when treated with 10^-6^ M synthetic germination stimulant, GR-24.

Biochars were mixed with a naturally fertile, clay-rich soil (55% clay, 25% silt, 20% sand, 2% organic matter, pH 7.2) from the north of Israel in a cement mixer at 0, 0.15, 0.3, 0.9, and 1.5% by dry weight. *P. aegyptiaca* seeds were mixed with the dry soil-biochar mixtures at an infestation rate of 15 mg seeds/kg soil mixture. Three control treatments were included: (i) no biochar and no *P. aegyptiaca* infestation; (ii) no biochar and *P. aegyptiaca* infested; and (iii) biochar at 1.5% and no *P. aegyptiaca* infestation.

Forty-day old tomato seedlings were transplanted into 2 L pots filled with the various soil mixtures and grown between April and July 2014 in a net house with irrigation as required. No additional nutrients or chemicals were added. The pots were arranged randomly in a single block. Sixty days after the seedlings were transferred, the soil was removed, tomato roots were washed, and tomato and *P. aegyptiaca* dry above-ground biomass were determined gravimetrically after drying to a constant weight at 60°C. *P. aegyptiaca* attachments were gently removed from the tomato roots and counted. A repeat experiment was conducted between July and October 2014.

The experimental design and statistical analyses were carried out according to Onofri et al. (2010). Experiments were arranged in a two-factorial design (biochar type and concentration) with six replications, and subjected to ANOVA testing. Because of non-homogeneity of the variance, data were log transformed. There was no significant experiment by treatment interaction, therefore, results of the two repeat experiments were combined and analyzed as 12 replications for each treatment. Means comparison was carried out by Tukey–Kramer Honestly Significant Difference (HSD) test at an α level of 0.05 computed by JMP software (version 7).

### Split Root Experiment in Polyethylene Bags

**FIGURE 1:**
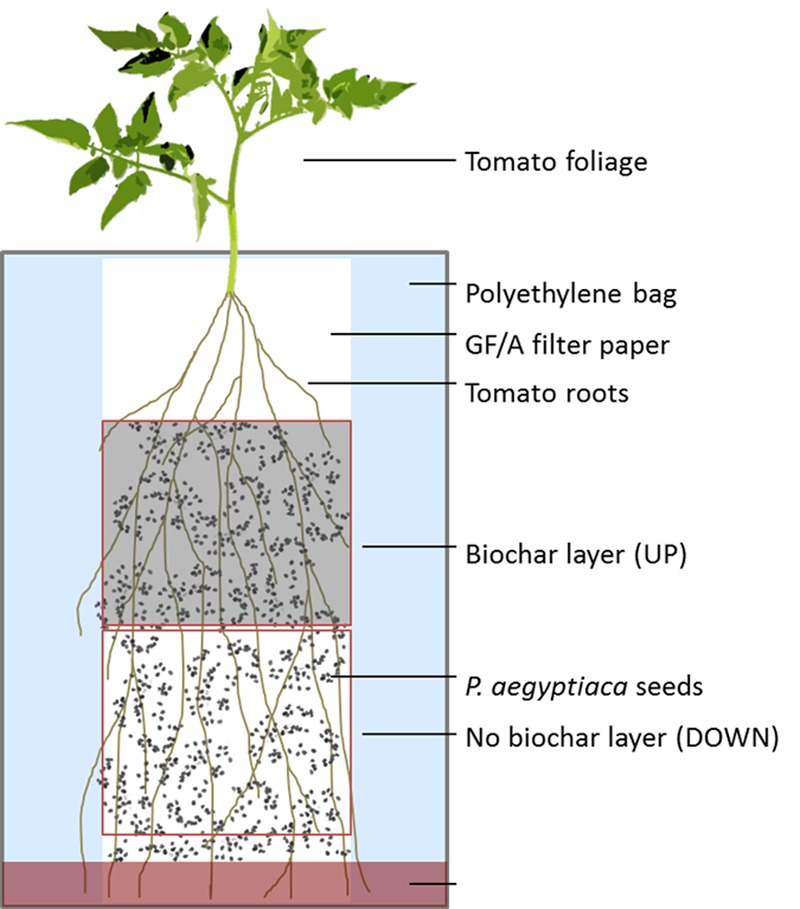
Schematic representation of the split-root polyethylene bag experimental design.

Tomato seedlings were cultivated in polyethylene bags (PEB) in a split-root type of arrangement that enabled evaluation of whether biochar had any systemic impact on seed germination or radicle attachment to the root. The PEBs were prepared according to Parker and Dixon (1983) and modified as required for this study. The root systems of 30-day-old tomato plants were cut and the stems re-rooted in sterile water for 7 days. *P. aegyptiaca* seeds were surface-sterilized for 3 min in 70% ethanol followed by 1% sodium hypochlorite for 10 min, and were then washed four times with sterile water. The upper half of a 10 cm × 22 cm sheet of glass fiber filter paper (Whatman, GF/A, Whatman International Ltd., Maidstone, England) was uniformly covered with 5 mL of a well-mixed biochar (GHW-350)/water suspension at concentrations of 0, 0.05, and 0.1% (w/v). One re-rooted plant was mounted onto each prepared filter paper, with the upper part of the root zone on the biochar-containing portion of the filter, and the lower part of the root zone on the biochar-free portion of the filter. Sterilized *P. aegyptiaca* seeds were then evenly scattered across the filter paper at a concentration of 50 seeds per cm^2^. The plant-containing sheets were inserted into a clear polyethylene bag to which 20 ml of sterilized half-strength Hoagland nutrient solution (Hoagland and Arnon, 1950) was added. The experimental setup is depicted schematically in **Figure 1**. The PEBs were placed in a growth chamber under a 16/8 h day/night condition at 24°C and were nourished twice weekly from the top of the bag with 20 to 30 ml half strength Hoagland nutrient solution as required.

After 14 and 21 days, a binocular microscope (DZ, Zeiss, Germany) at 40× magnification was used to detect seed germination and root attachment, respectively. The percentage of germinated seeds and number of radicle attachments along the main tomato root were recorded.

Two identical experiments were performed in series; in both, the PEBs were arranged in completely randomized design in four replicates. No experiment–treatment interaction was detected; therefore, results of experiments were combined and analyzed as 8 replicates by 2-way ANOVA. Means comparison was carried out by Tukey–Kramer HSD test at an α level of 0.05 computed by JMP software (version 7).

### Activity of the Synthetic Stimulant GR-24 as Affected by Adsorption on Biochar

GR-24 is a standard artificial stimulant used to evaluate parasitic weed seed germination under laboratory conditions. Typically, the germination rate in response to GR-24 is higher than the germination rate in the natural environment. The ability of biochar to adsorb the seed stimulant molecule, GR-24, and hence interfere with seed germination, was evaluated using a *P. aegyptiaca* seed germination test. *P. aegyptiaca* seeds were surface-sterilized as before. About 50–70 seeds were scattered on the surface of a GF/A disk (9 mm diameter). A total of 48 disks were prepared, the disks were placed in sterile plastic Petri dishes (50 mm diameter) at 4 disks per dish (12 total dishes). Each disk was wetted with 32 μl sterile water. The Petri dishes were closed and then sealed with Parafilm and covered completely in aluminum foil; the seeds were thusly conditioned at 22°C for 7 days. Following the conditioning phase, the seed-bearing disks were blotted and transferred to new Petri dishes, 4 per dish, for subsequent treatment by the test solutions.

Sixteen different treatment solutions were prepared by adding finely ground biochar (GHW-350) at different levels (0, 0.005, 0.01, and 0.05% by weight) to solutions of GR-24 prepared in Millipore water at desired initial nominal GR-24 concentrations (10^-7^, 10^-8^, 10^-9^, and 10^-10^ M). The biochar/GR-24 water suspensions were kept at pH 7 and shaken at room temperature for 2 h, and then filtered using Millipore PVDF 0.2 μm filters to remove the biochar. By maintaining a neutral pH, any potential alkaline deactivation of the GR-24 stimulant was avoided. Aliquots (26 μl) of the filtered treatment solutions were applied to the preconditioned seed disks (4 per petri dish), the dishes were sealed and incubated as before. Each petri dish was arranged as a block containing one disk each of the four filtered test solutions (0, 0.005, 0.01, and 0.05% by weight); there were three replicate blocks for each GR-24 concentration.

*Phelipanche aegyptiaca* seed germination was quantified after 7 days exposure to the GR-24 solutions by counting total and germinated seeds under a binocular microscope. Two identical experiments were performed consecutively, both arranged in block design in three replicates. No experiment–treatment interaction was detected; therefore, results of experiments were combined and analyzed as 6 replications by 2-way ANOVA in blocks. Means comparison was carried out by Tukey–Kramer HSD test at an α level of 0.05, computed by JMP software (version 7).

## Results

### Pot Trials

**FIGURE 2:**
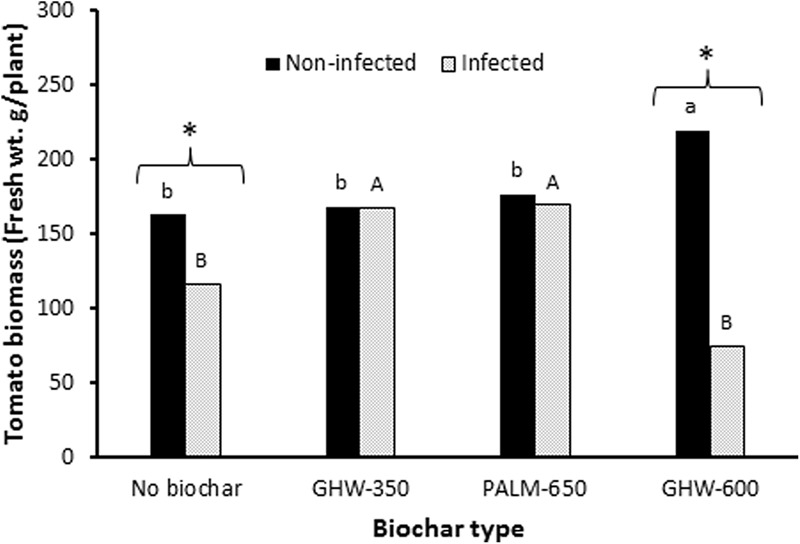
Effect of biochar type (*X*-axis) and *P*. *aegyptiaca* (Egyptian broomrape) infection on tomato plant biomass (*Y*-axis). Histogram bars give the mean tomato plant wet weight in g per plant at biochar dose of 1.5 wt%. Means labeled by different lowercase letters (non-infected) are significantly different according to the Tukey HSD test (α = 0.05). Means labeled by different uppercase letters (infected) are significantly different according to the Tukey HSD test (α = 0.05). The asterisk (^∗^) indicates a significant difference at α = 0.05 between the biomass of infected and non-infected tomato plants within a given biochar treatment.

Under non-infested conditions, the addition of GHW-600 biochar at 1.5% resulted in a significant increase in tomato plant biomass as compared with the no-biochar, non-infested control (**Figure 2**). The addition of the other two biochars (GHW-350 and PALM-650) at 1.5% had no significant impact on tomato plant growth compared with the no-biochar, non-infested control (**Figure 2**).

Under *P. aegyptiaca* infestation, additions of GHW-350 and PALM-650 biochars resulted in increases in tomato plant biomass as compared with the no-biochar, infested control (**Figure 2**). In contrast, addition of the GHW-600 biochar did not improve tomato plant growth compared with the no-biochar, infested control treatment (**Figure 2**).

**FIGURE 3:**
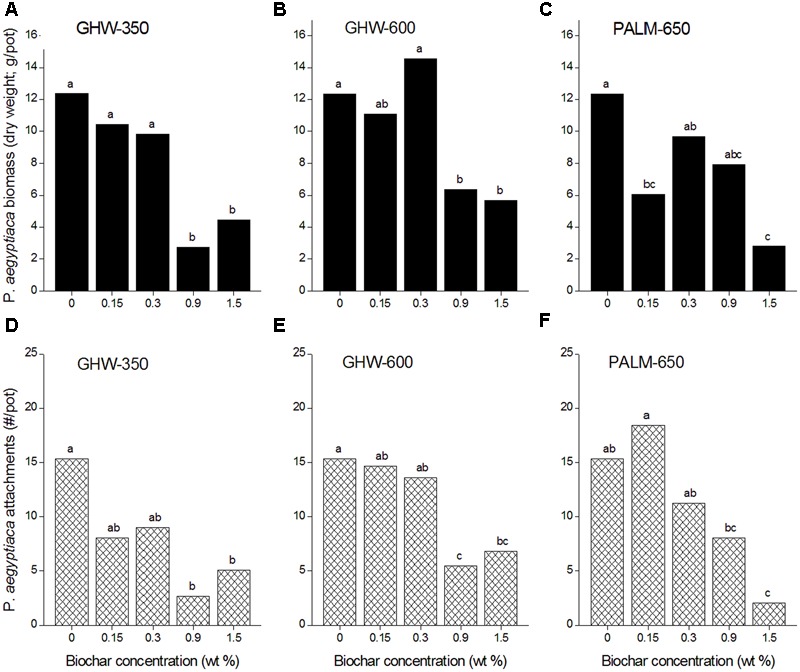
*Phelipanche aegyptiaca* (Egyptian broomrape) infection parameters [biomass in dry weight in g/pot, **(A–C)**, and attachments in number/pot, **(D–F)**] versus biochar concentration (wt%) for the three different biochars. Experiments were arranged in a two-factorial design (biochar type and concentration) with six replications and subjected to ANOVA testing. Means comparison was carried out by Tukey–Kramer Honestly Significant Difference (HSD) test at an α value of 0.05 computed by JMP software (version 7). Means labeled by different letters are significantly different according to the HSD test.

Compared with the no-biochar controls, all three biochars added at 1.5% caused a reduction in *P. aegyptiaca* biomass (**Figures 3A–C**), while both GHW biochars also reduced *P. aegyptiaca* biomass when added to the soil at 0.9% (**Figures 3A,B**). *P. aegyptiaca* attachments were reduced at 0.9 and 1.5% biochar doses for all three biochar types compared with the no-biochar controls (**Figures 3D–F**).

**FIGURE 4:**
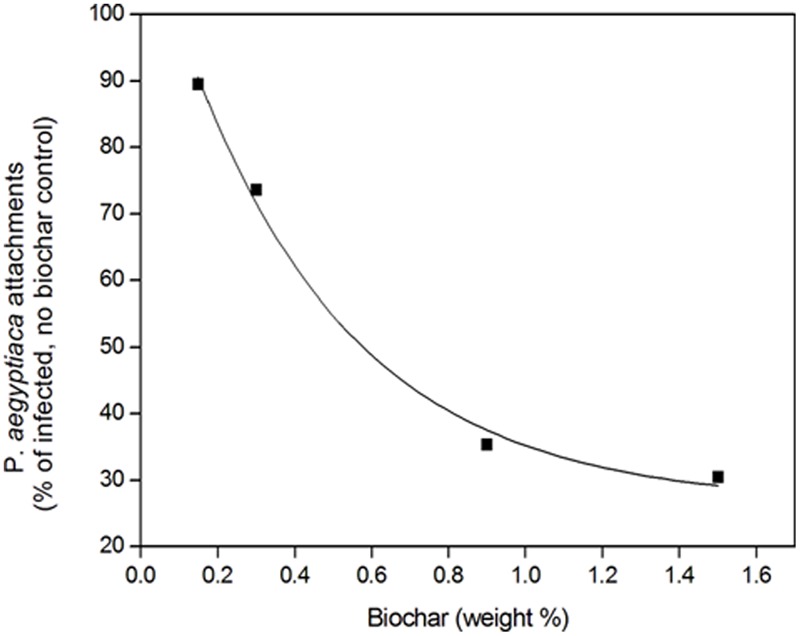
*Phelipanche aegyptiaca* (Egyptian broomrape) attachments (number/pot) in the root zone relative to the no-biochar, infested control as a function of biochar dose. Results for the three biochar types are pooled. The fitted line is a first order exponential decay curve of the form y = y_0_ + Ae^-x/t_1_^, where y_0_ is 26.4, *A* is 91.1, and *t*_1_ is 0.43, *R*^2^ = 0.995.

**FIGURE 5:**
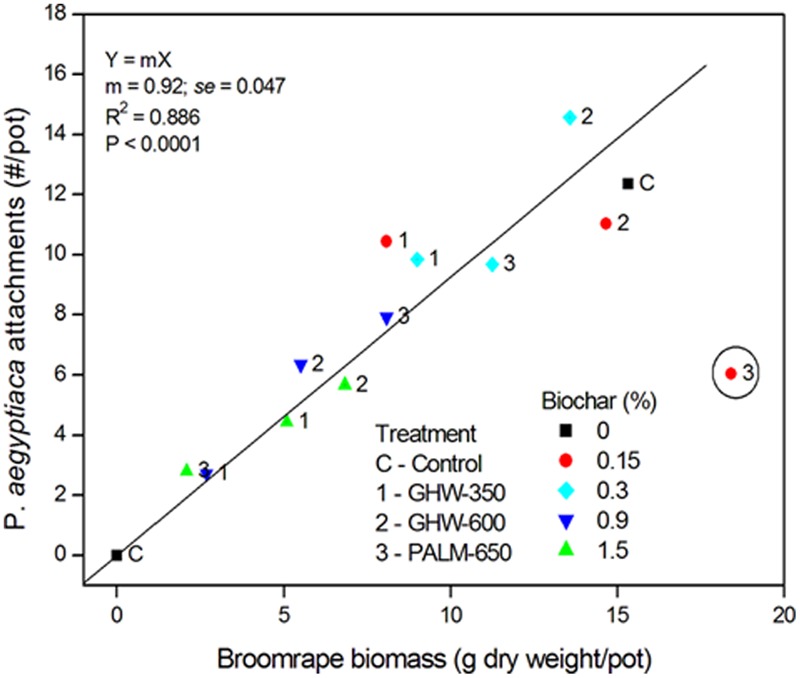
*Phelipanche aegyptiaca* (Egyptian broomrape) attachments (number/pot) versus *P*. *aegyptiaca* biomass (dry weight in g/pot). Biochar concentrations are depicted in symbols of different colors and shapes (as given in legend), and each data point is labeled according to its biochar type (given in legend). One outlier (encircled red dot labeled with the number 3) is excluded from the regression.

A negative relationship between number of attachments and biochar dose, pooled for all three biochars, was revealed (**Figure 4**). The higher biochar doses (0.9 and 1.5% by weight) effected a reduction in number of attachments to circa 35% of the number in the biochar-less control. Moreover, a positive linear relationship between number of attachments and *P. aegyptiaca* biomass was detected, with higher biochar dose treatments having fewer attachments and lower biomass (**Figure 5**). There was no general trend in efficacy amongst the three biochars (**Figure 5**).

### Split-Root PEB Experiments

**FIGURE 6:**
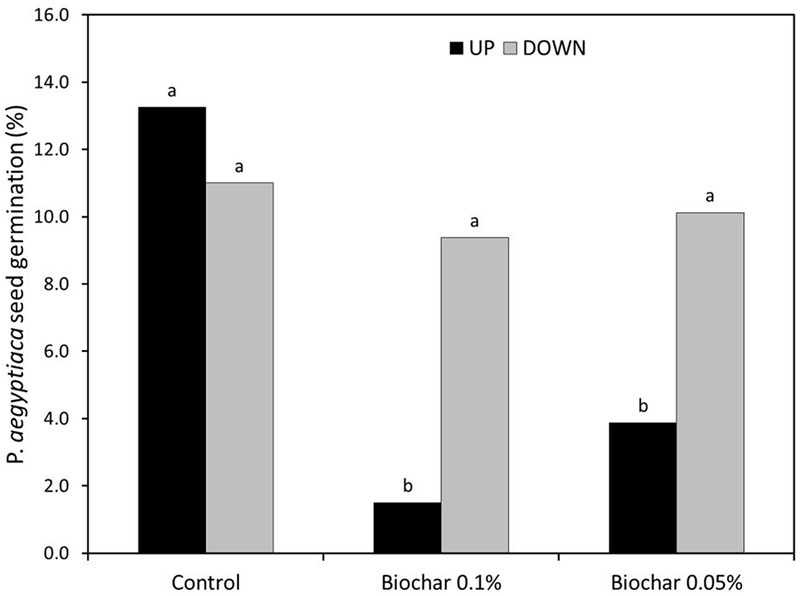
Split-root experimental results. ‘UP’ signifies the upper portion of the root zone and ‘DOWN’ signifies the bottom portion of the root zone. In the Control treatment, neither UP nor DOWN were exposed to biochar. In the two biochar treatments (Biochar 0.1% and biochar 0.05%), the UP part was exposed to the specified concentration of biochar; the DOWN part was free of biochar. The *Y*-axis gives the percentage (%) of *P*. *aegyptiaca* (Egyptian broomrape) seeds that germinated. Results of two experiments were combined and analyzed as 8 replicates by 2-way ANOVA. Means comparison was carried out by Tukey-Kramer Honestly Significant Difference (HSD) test at an α level of 0.05 computed by JMP software (version 7). Means labeled by different letters are significantly different according to the HSD test.

**FIGURE 7:**
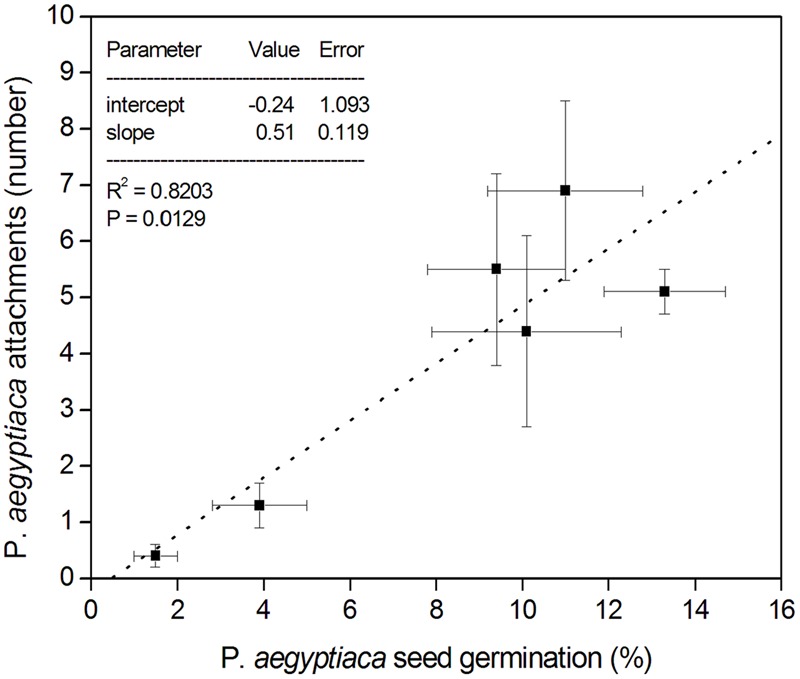
Split-root experimental results. The *X*-axis presents the percentage (%) of *P*. *aegyptiaca* (Egyptian broomrape) seeds that germinated in both UP and DOWN portions of all three treatments; the *Y*-axis presents the number of attachments of *P*. *aegyptiaca* in both UP and DOWN portions of all three treatments. Error bars in both *X*- and *Y*-axis directions denote the standard error of the mean, represented by the data points.

In the split root experiment, the percentage of *P. aegyptiaca* seeds that germinated was the same in both the upper and lower parts of the negative control treatment (no biochar on either upper or lower parts; **Figure 6**). Moreover, the germination percentage in the negative control was indistinguishable from that in the no-biochar lower parts of the test treatments (**Figure 6**). In the biochar-exposed upper parts, however, the germination percentage was strongly reduced compared with that in the no-biochar lower parts, and as compared with the negative control (no biochar on either upper or lower part; **Figure 6**). This same effect was observed at both levels of biochar addition (**Figure 6**). The number of radicle attachments (parasites per plant) was linearly related to the seed germination percentage across the whole root, including biochar-exposed and biochar-free parts (**Figure 7**). This linear relationship demonstrates that the biochar was not toxic to the sensitive radicle. The results also demonstrate there was no direct or systemic effect of biochar on the penetrability of the root membrane. Together, these results show that addition of biochar reduced seed germination, and it is the reduction in seed germination that caused the reduction in number of radicle attachments.

### Effect of GR-24 Sorption on Biochar on Germination of *P. aegyptiaca* Seeds

**FIGURE 8:**
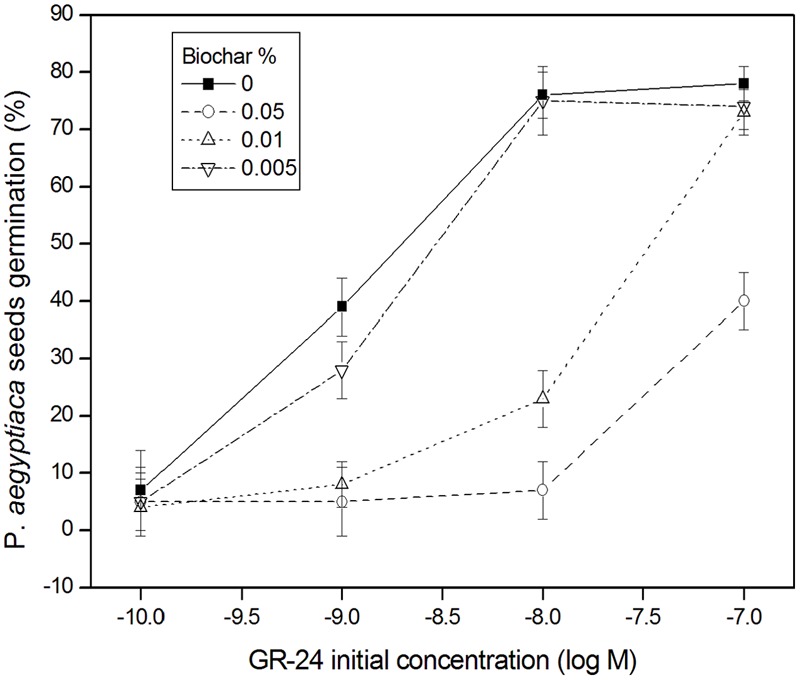
*Phelipanche aegyptiaca* (Egyptian broomrape) seed germination percentage (*Y*-axis) as a function of GR-24 initial concentration (M) (*X*-axis) at different additions of GHW-350 biochar (%); mean and standard error (bars) are presented. Least significant difference (LSD) values (in %) based on the Tukey-Kramer HSD test (*P* ≤ 0.05) are as follows: for GR24 concentration of 10^-10^ = 6.9; 10^-9^ = 11.4; 10^-8^ = 10.2; 10^-7^ = 14.4.

In the no-biochar control solutions of GR-24, the maximally effective concentrations of GR-24 were 10^-8^ and 10^-7^ M (78% of *P. aegyptiaca* seeds germinated; **Figure 8**). In the GR-24 solutions to which biochar had been added at the two highest doses (0.01 and 0.05% biochar), *P. aegyptiaca* seed germination was substantially reduced compared with the no-biochar control solutions (**Figure 8**). Indeed, at the highest biochar dose (0.05%), germination percentage was decreased by the equivalent of two orders of magnitude in GR-24 concentration. This is apparent in **Figure 8**, where the same percentage of *P. aegyptiaca* seeds germinated in the solution having an initial concentration of 10^-7^ M GR-24 plus 0.05% biochar, as in the solution having an initial concentration of 10^-9^ M GR-24 and no biochar. Even the lowest biochar dose, 0.005%, resulted in a decrease in *P. aegyptiaca* seed germination by about 25% at an initial concentration of GR-24 of 10^-9^ M in comparison with the no-biochar added control. Insomuch as the pH in all the test solutions was held at a constant neutral value, it is apparent that the stimulant molecule was not alkaline-deactivated. The results show that reduced germination of *P. aegyptiaca* seeds in biochar-treated GR-24 solutions is a result of reduced GR-24 solution concentration due to its adsorption on the biochar.

## Discussion

In sum, the results shows that biochar added to the soil can reduce the extent of *P. aegyptiaca* infection in tomato, mainly by reducing *P. aegyptiaca* seed germination due to adsorption of the stimulant molecule on the biochar. The reduction in seed germination leads to a decrease in *P. aegyptiaca* attachments, which in turn leads to a decrease in *P. aegyptiaca* biomass. What is more, some types of biochar can contribute to improved tomato plant growth and development even in the event of *P. aegyptiaca* infestation. It was found that all three tested biochars exhibited similar abilities to reduce *P. aegyptiaca* attachments and biomass, which reflects their essential similarity in the physical and chemical attributes that are important for sorption, namely specific surface area and iodine number (**Table 1**).

In theory, there are a number of ways by which biochar could interfere with the germination of root parasitic weed seeds such as broomrape and *Striga* spp.: (1) Physically adsorb signaling molecules, preventing them from reaching the seeds; (2) Chemically inactivate the signaling molecules; (3) Release chemicals that are toxic to the seeds or interfere with stimulant receptor recognition; (4) Induce reduction in host production or exudation of the germination-signaling molecule; (5) Promote changes in the rhizosphere microbiome that affect root parasitic weed seed germination; and (6) Combinations of these factors. However, all but the first of these possible mechanisms can be eliminated from consideration in view of the results reported herein.

One of the outcomes of the split-root experiment is that biochar had no effect on the host production or exudation of the signaling molecule(s). This can be understood by virtue of the fact that in the no-biochar part of the split biochar/no-biochar systems, the extents of germination and attachment were identical to those in the negative control (no part of the root system exposed to biochar). Moreover, the linear relationship between germination percentage and number of radicle attachments across all the studied biochar concentrations (0, 0.05, and 0.1%) shows the biochar was not toxic to the radicle. Given that broomrape radicles are generally more sensitive to toxins than are broomrape seeds (Timko and Scholes, 2013), it can be assumed that the biochar also was not toxic to the seeds. It is also apparent from these results that the biochar had neither a systemic nor contact effect on the integrity of the root membrane.

Some rhizosphere microorganisms can affect root parasitic weed seed germination by competing for the signaling molecules, for example, arbuscular mycorrhizal fungi (AMF). Many of the compounds that activate root parasitic weed seed germination (such as flavonoids, sesquiterpenes, and strigolactones) are similar to or the same as those that stimulate growth and branching in AMF (Akiyama et al., 2005; Bucher et al., 2009). Biochar additions to soil are known to affect AMF abundance, and it was recently suggested that this is due to biochar adsorption of inhibitory or signaling compounds (Thies et al., 2015). While levels of microorganisms such as AMF that can compete with weed seeds for exuded stimulant molecules were not determined in these experiments, it is known that AMF do not develop in well-nourished semi-sterile hydroponic systems such as those employed (Gomez-Roldan et al., 2008). This means there is little likelihood that microorganisms such as AMF out-competed the seeds for the germination stimulant molecule.

The active chemical moiety of the synthetic stimulant GR-24 is the lactone ring, which can open at elevated pH and lose its stimulant activity (Yoneyama et al., 2013). However, pH was maintained at 7 in the adsorption/germination experiment. Thus, the totality of the experiments points toward physical adsorption of the signaling molecules by the biochar as being the major factor responsible for the reduction in germination of the root parasitic weed seeds.

Adding biochar to soil represents the first-ever ecological approach to root parasitic weed management. It is also suitable for organic agriculture. The potential of this discovery may be far-reaching. For example, the use of biochar may make it unnecessary to develop root parasitic weed resistant crops such as those based on the absence of *in planta* production of germination stimulant chemicals (Dor et al., 2009), particularly as such mutant plants often have irregular branching and growth patterns. It is possible that adding biochar can help offset temperature-dependent losses of natural resistance observed in various broomrape resistant varieties (Eizenberg et al., 2003). Reducing broomrape germination and parasitism by using biochar could potentially minimize the development of new virulent broomrape races (Pérez-de-Luque et al., 2009).

It is worthwhile noting that the use of biochar for ecological weed control will be effective only for root parasitic weeds that need chemical stimulation for germinating their seeds. When a stimulant is not required for seed germination, such as for autotrophic plants, adding biochar has not been an effective approach for weed control (Major et al., 2005). This is because, like with other non-parasitic plants, biochar mainly has a neutral or stimulating effect on autotrophic weed growth. Nor was biochar found to be an effective treatment for reducing the biomass of the hemiparasitic yellow rattle weed (Smith and Cox, 2014). The yellow rattle also does not require a host-derived stimulant molecule for germination of its seeds.

## Conclusion

The finding that small additions of various biochars to soil can significantly reduce infection by the root parasitic weed, *P. aegyptiaca*, in tomato, portends an innovative means of ecological control over such pests and justifies testing additional host-parasite systems under horticultural and field conditions. The major mechanism responsible for biochar interference with parasitic weed seed germination is adsorption of the stimulant molecule. Hence, it will be straightforward to design biochars having high adsorption capacities specifically for this application. To date, the pyrolysis/biochar platform is widely viewed as a potentially important tool for global climate change mitigation; however, it still does not enjoy widespread implementation, in part because the benefits of biochar addition to soil are not yet well-understood, and in part because the costs of biochar use in agriculture are still too high. The ability of biochar to decrease infection by parasitic weeds in important crops may change treatment strategies for this family of parasites, and also enhance the economic feasibility of biochar use in agriculture, and as a result, of the pyrolysis/biochar platform in its entirety.

## Author Contributions

HE was the lead designer of the experiments, and an integral part of result analysis and interpretation, drafting, revising and approving the manuscript, and is accountable for the accuracy of the reported experimental data. DP was responsible for performing the split-root and seed germination experiments, HZ was responsible for performing the pot trials, and LT was responsible for performing the physical and chemical characterization of the biochars. DP, HZ, and LT acquired and analyzed the data for which they were responsible, are accountable for its accuracy, and reviewed the manuscript for accuracy. EG was responsible for conceiving the research project, results analysis and interpretation, drafting, revising and approving the manuscript, and is accountable for the data reported inside.

## Conflict of Interest Statement

The authors declare that the research was conducted in the absence of any commercial or financial relationships that could be construed as a potential conflict of interest.

## Footnotes

1 www.blucomb.com
